# OK-432 Acts as Adjuvant to Modulate T Helper 2 Inflammatory Responses in a Murine Model of Asthma

**DOI:** 10.1155/2018/1697276

**Published:** 2018-10-08

**Authors:** Cheng-Jang Wu, Pin-Hsun Tseng, Cheng-Chi Chan, Sara Quon, Li-Chen Chen, Ming-Ling Kuo

**Affiliations:** ^1^Chang Gung Molecular Medicine Research Center, Chang Gung University, Taoyuan, Taiwan; ^2^Graduate Institute of Biomedical Sciences, College of Medicine, Chang Gung University, Taoyuan, Taiwan; ^3^Division of Biological Sciences, University of California, San Diego, La Jolla, CA 92093, USA; ^4^Division of Allergy, Asthma, and Rheumatology, Department of Pediatrics, Chang Gung Memorial Hospital, Taoyuan, Taiwan; ^5^Department of Microbiology and Immunology, Graduate Institute of Biomedical Sciences, College of Medicine, Chang Gung University, Taoyuan, Taiwan; ^6^Chang Gung Immunology Consortium, Chang Gung Memorial Hospital and Chang Gung University, Taoyuan, Taiwan; ^7^Research Center for Chinese Herbal Medicine, College of Human Ecology, Chang Gung University of Science and Technology, Taoyuan, Taiwan

## Abstract

Enhanced type 2 helper T (Th2) cell responses to inhaled harmless allergens are strongly associated with the development of allergic diseases. Antigen formulated with an appropriate adjuvant can elicit suitable systemic immunity to protect individuals from disease. Although much has been learned about Th1-favored immunomodulation of OK-432, a streptococcal preparation with antineoplastic activity, little is known about its adjuvant effect for allergic diseases. Herein, we demonstrate that OK-432 acts as an adjuvant to favor a systemic Th1 polarization with an elevation in interferon- (IFN-) *γ* and ovalbumin- (OVA-) immunoglobulin (Ig) G2a. Prior vaccination with OK-432 formulated against OVA attenuated lung eosinophilic inflammation and Th2 cytokine responses that were caused by challenging with OVA through the airway. This vaccination with OK-432 augmented the ratios of IFN-*γ*/interleukin- (IL-) 4 cytokine and IgG2a/IgG1 antibody compared to the formulation with Th2 adjuvant aluminum hydroxide (Alum) or antigen only. The results obtained in this study lead us to propose a potential novel adjuvant for clinical use such as prophylactic vaccination for pathogens and immunotherapy in atopic diseases.

## 1. Introduction

Bronchial asthma is a worldwide public health problem increasing in incidence, morbidity, and mortality during the last three decades [1, 2]. The cardinal features of asthma, which are bronchial eosinophil infiltration, airway-hypersensitivity, elevated serum IgE levels, mucus hypersecretion, and airway remodeling, are strongly associated with enhanced Th2 cell responses to inhaled harmless allergens [3]. As such, Th2 cells play a critical role in the induction and exacerbation of allergic asthma. Th2 cytokines such as IL-4 and IL-13 induce IgE synthesis in B cells [4, 5]. Another Th2 cytokine, IL-5, regulates the homeostasis of eosinophils, which largely contributes to immunopathology [6]. Cross-linking of allergen-specific IgE bound to high-affinity IgE receptors (Fc*ε*R) on mast cells and eosinophils with reencountered allergens results in cell degranulation, the release of soluble mediators, such as histamine and leukotrienes, and the exacerbation of the disease [7].

The current therapies for asthma, consisting mainly of inhalers with corticosteroids and long-acting *β*2-agonists, are relatively safe and cheap [8]. However, asthma remains poorly controlled in more than half of afflicted patients [9]. Novel preventive strategies capable of modulating Th2 responses might therefore be beneficial, since the induction of antigen-specific Th1 responses has shown promising efficacy by balancing Th2-mediated allergic diseases [10]. Immunotherapy using Th1-bias adjuvants to attenuate Th2 immune responses to the injected allergens has shown promising clinical results [11]. It has also been shown that bacterial components existing at the site of sensitization suppress Th2 allergic responses [12, 13]. As a result, exploring novel adjuvant that skews Th2-associated allergic responses toward a protective Th1 cytokine profile is of great interest.

OK-432 (trade name Picibanil) is a streptococcal preparation with strong anticancer activity [14]. Streptococcus pyogenes (group A) strain Su is treated with penicillin G and heated to increase the antitumor activity and remove its toxic potency prior to lyophilization [15]. It has been shown that OK-432 is a potent inducer of IL-12 and subsequently induces IFN-*γ* production in both human and mouse cells [16]. Clinically, OK-432 is approved as an anticancer agent in Japan and has been extensively administered against malignant tumors with a good safety profile [17–19]. Although much has been learned over the past few decades about Th1-favored immunomodulation of OK-432, little is known about its adjuvant effect in immunotherapy of allergic diseases.

In the present study, we have demonstrated that OK-432 acts as an adjuvant to enhance strong antigen-specific Th1 responses in mice relative to Th2 adjuvant aluminum hydroxide as seen by its favoring of systemic Th1 polarization with an elevation in IFN-*γ* and OVA-IgG2a. Mice immunized with OK-432 formulation against chicken OVA were promisingly more resistant to lung eosinophilic inflammation and Th2 cytokine responses when challenged with the same antigen through the airway. OK-432 immunization resulted in increased ratios of IFN-*γ*/IL-4 cytokine and IgG2a/IgG1 antibody. Our findings demonstrate that OK-432 can be used as an adjuvant to promote potent Th1 and suppress Th2 for the development of efficacious allergen immunotherapy.

## 2. Materials and Methods

### 2.1. Animals and Reagents

Five-week-old male C57BL/6 mice were purchased from the National Laboratory Animal Center, Taipei, Taiwan. Male mice were used only to avoid the indirect immunological effects from female sex hormones. Mice were maintained and handled according to the guidelines of the Animal Care Committee of Chang Gung University, NIH Guidelines for the Care and Use of Laboratory Animals, and the ARRIVE guidelines. Animal procedures have been approved by the Animal Care Committee of Chang Gung University. OK-432 (Picibanil®) was purchased from Chugai Pharmaceutical Ltd.

### 2.2. Immunization and Challenge Protocol

Eight-week-old mice were immunized by intraperitoneal (i.p.) injection of 50 *μ*g ovalbumin (OVA, grade V, Sigma-Aldrich Inc., MO, USA), 50 *μ*g OVA mixed with 0.8 mg aluminum hydroxide (Pierce Biotechnology Inc., Rockford, IL, USA), or 50 *μ*g OVA mixed with 0.5 KE (Klinische Einheit: clinical unit; 1 KE equals 0.1 mg lyophilized streptococci) OK-432. All reagents for immunization were prepared in 200 *μ*l PBS. On day 28, these mice were boosted by another i.p. injection with OVA formulated with the corresponding adjuvant. All mice were sacrificed on day 35. Serum and spleen were collected for antigen-specific antibodies and cytokine assays. In the challenge model, the immunized mice were further challenged with 10 *μ*g OVA in 50 *μ*l PBS on days 35, 36, and 37 by oropharyngeal instillation (o.i.). The pulmonary function test (PFT) was analyzed on day 38, and mice were sacrificed the next day.

### 2.3. Pulmonary Function Test

After the last challenge, airway resistance of mice was analyzed by stimulating with normal saline and increasing concentrations of methacholine (Sigma-Aldrich; 10, 30, and 100 mg/ml) for 30 seconds. Mice were then placed in the detection chamber (SFT3812) linked to the sensor, and the resistance index (RI) was recorded for 3 minutes. These data were calculated and analyzed by BuxcoFinePointe™ RC. All devices were from Buxco Electronics.

### 2.4. Bronchoalveolar Lavage (BAL) Fluid

The tracheas of mice were surgically cannulated using a 24G I.V. catheter, and the lungs were lavaged three times with 1 ml PBS containing 0.1 mM EDTA. Total cells of BALF were collected, and the absolute numbers were counted using trypan blue dye exclusion. For differential cell counts, BAL cells were cytocentrifuged (Shandon Cytospin 4; Thermo Fisher Scientific, Pittsburg, PA, USA) and stained with Liu staining solution as previously described [12]. Differential cell counts were based on the staining morphology profiles of 200 cells per sample.

### 2.5. Histology

To assess immunopathology, lung tissues were removed and immediately fixed in 10% formalin solution. Sections were embedded in paraffin and stained for hematoxylin and eosin (H&E). The percentages of cell infiltration (defined as the inflammatory index) were analyzed by MetaMorph microscopy automation and image analysis software (Molecular Devices, Sunnyvale, CA). Data were analyzed from three sections of each mouse lung and five selected areas with 100 *μ*m square size in each section. The data were calculated using the mean values of sensitized control mice as 100%.

### 2.6. RNA Isolation and Quantitative RT-PCR

For detecting cytokine levels in sensitized lungs, the total lung RNA was extracted by using TRIzol reagent (Invitrogen, Life technologies) and treated with DNase I (Fermentas, Thermo Fisher Scientific). Complementary DNAs (cDNAs) were generated by M-MLV reverse transcriptase (Invitrogen) using random primers, and real-time PCR was performed using SYBR Green PCR kits (Fermentas). They were then amplified via a CFX Connect real-time PCR detection system (Bio-Rad). The thermal cycling conditions were as follows: 95°C for 10 min and 40 repeats at 95°C for 15 sec and 60°C for 1 min. The relative expression of each gene was calculated by normalizing with housekeeping gene *β*-actin. Primers are as follows: IL-4—5′-GGTCTCAACCCCCAGCTAGT-3′ (F) and 5′-GCCGATGATCTCTCTCAAGTGAT-3′ (R); IL-5—5′-CTCTGTTGACAAGCAATGAGACG-3′ (F) and 5′-TCTTCAGTATGTCTAGCCCCTG-3′ (R); IL-13—5′-CCTGGCTCTTGCTTGCCTT-3′ (F) and 5′-GGTCTTGTGTGATGTTGCTCA-3′ (R); IFN-*γ*—5′-ATGAACGCTACACACTGCATC-3′ (F) and 5′-CCATCCTTTTGCCAGTTCCTC-3′ (R); CCL11—5′-GAATCACCAACAACAGATGCAC-3′ (F) and 5′-ATCCTGGACCCACTTCTTCTT-3′ (R); and *β*-actin—5′-GGCTGTATTCCCCTCCATCG-3′ (F) and 5′-CCAGTTGGTAACAATGCCATGT-3′ (R).

### 2.7. Measurement of Serum Antibody Levels

The titers of total IgE antibodies in the serum were determined using ELISA (Mouse IgE Ready-SET-Go, Affymetrix eBioscience, Thermo Fisher Scientific) according to the manufacturer's instructions. OVA-specific IgG1, IgG2a, and IgE antibody levels were measured by ELISA as described previously [20]. A 96-well plate (Costar, Corning, NY, USA) was coated with 10 *μ*g/ml OVA at 37°C for one hour. Serum samples were diluted in blocking buffer (PBS containing 1% bovine serum albumin) and incubated in OVA-coated plates at 37°C for one hour. Next, biotinylated rat anti-mouse IgG1 (A85-1), IgG2a (R19-15), and IgE (R35-118) monoclonal antibodies (all from BD PharMingen, San Diego, CA, USA) were added into the plates and incubated at 37°C for one hour. Streptavidin-conjugated horseradish peroxidase (HRP) (BD PharMingen) was added to the plates and incubated at room temperature for 30 minutes in the dark. Finally, substrate solution (TMB, BD Biosciences) was added to the plates, and the reaction was stopped after 20 minutes with 2 N H_2_SO_4_. Absorbance was measured by an ELISA reader (Sunrise, TECAN, Australia) at 450 nm.

### 2.8. Cell Culture and Cytokine Assays

After the mice were sacrificed, spleens were removed and gently grounded in sterile PBS. A mesh filter was used to obtain single cell suspensions, and red blood cells were depleted by using RBC lysing buffer. Splenocytes (5 × 10^6^ cells/ml) were cultured with RPMI medium (Gibco, Life Technologies, Grand Island, NY, USA) containing 10% fetal bovine serum (FBS, Gibco) and 1% penicillin/streptomycin (Gibco) and were stimulated by 100 *μ*g/ml OVA in 24-well plates (Costar) at 37°C under 5% CO_2_ in a humidified incubator. The supernatants were collected on day 6 for cytokine ELISA. The concentrations of interleukin- (IL-) 4, interferon-*γ* (IFN-*γ*) (DuoSet ELISA kit, R&D Systems), and IL-5 (BD OptEIA set, BD Pharmingen) were determined by ELISA according to the manufacturer's instructions. Cytokine concentrations were calculated based on a standard run in parallel with recombinant cytokines. The limits of detection were 15.6 pg/ml for IL-4 and 31.3 pg/ml for IL-5 and IFN-*γ*.

### 2.9. Statistical Analysis

Data were analyzed using the Mann–Whitney *U* test. Results are expressed as the mean ± standard error of the mean (SEM). *p* values of <0.05 were considered statistically significant.

## 3. Results

### 3.1. OK-432 Acts as Adjuvant to Induce Th1-Bias Immune Responses

**Figure 1:**
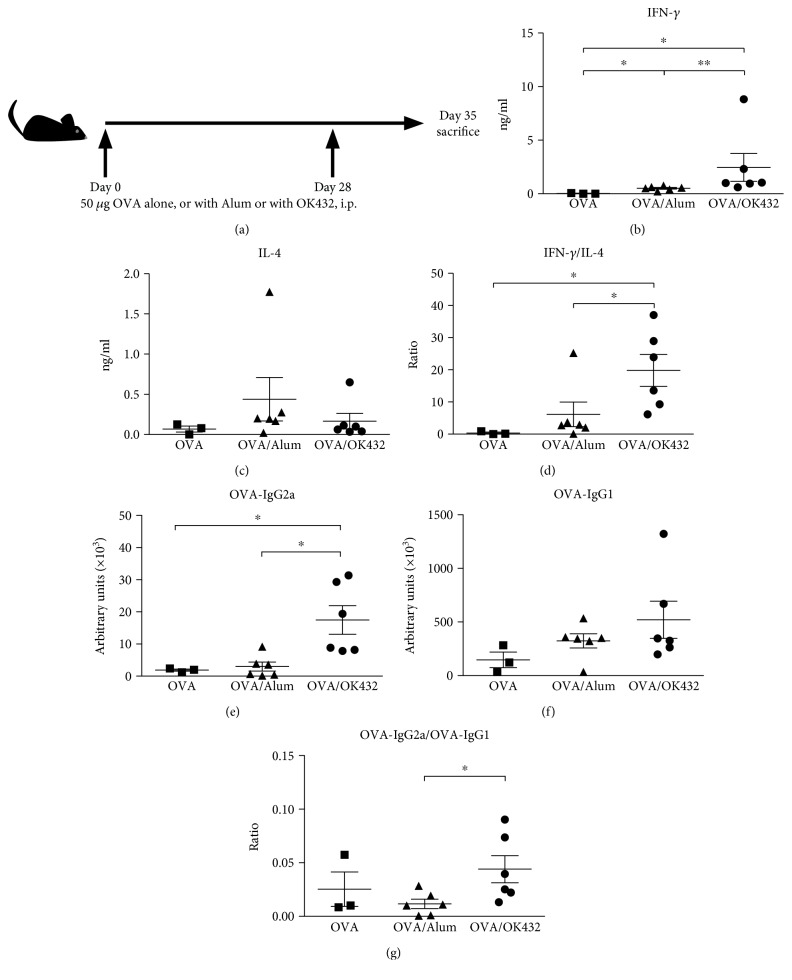
OK-432 is a strong Th1 adjuvant. (a) Schematic diagram depicting protocols for immunization protocol. The immune responses of B6 mice immunized with different formulations were determined. The levels of IFN-*γ* (b) and IL-4 (c) in culture supernatants, in which spleen cells were stimulated by 100 *μ*g/ml OVA for 6 days, and the titers of OVA-specific IgG2a (e) and IgG1 (f) in sera were measured by ELISA. The ratios of Th1/Th2 cytokine (d) and immunoglobulin (g) are shown. Data are represented as the mean ± SEM from two independent experiments (*n* = 3–6 per group). ^∗^*p* < 0.05; ^∗∗^*p* < 0.01.

To determine whether OK-432 can enhance antigen- (Ag-) specific immune responses, we first examined the consequences of B6 mice systemically immunized with either OVA alone (OVA) or OVA formulated with OK-432 (OVA/OK-432) (Figure 1(a)). The strong Th2 adjuvant aluminum hydroxide (Alum) was used as a parallel control (OVA/Alum). One month after immunization, mice were boosted with a second dose of the same corresponding formula. One week after boosting, OVA-specific humoral and cellular responses were measured. As shown in Figure 1(b), the amount of IFN-*γ* in the OVA-stimulated splenocyte supernatant was significantly higher in mice injected with OK-432-formulated OVA compared to Alum-formulated OVA or OVA alone. OK-432 also induced higher IFN-*γ*/IL-4 cytokine ratios than either no adjuvant or aluminum hydroxide (Figure 1(d)). In addition, the titers of OVA-specific IgG2a, a Th1 cytokine-regulated isotype, as well as the ratio of IgG2a/IgG1 were greatly enhanced in mice treated with OK-432 relative to those treated with Alum (Figures 1(e) and 1(g)). However, the levels of IL-4 in splenocyte supernatant and IgG1 in the sera of OVA/OK-432 mice were comparable or lower than those of OVA/Alum mice (Figures 1(c) and 1(f)). Our results have demonstrated the adjuvant effect of OK-432 to augment Th1-bias response by stimulating OVA-specific IgG2a and IFN-*γ* production.

### 3.2. OK-432 Adjuvant Controls Airway Inflammation in a Local OVA-Challenge Model

**Figure 2:**
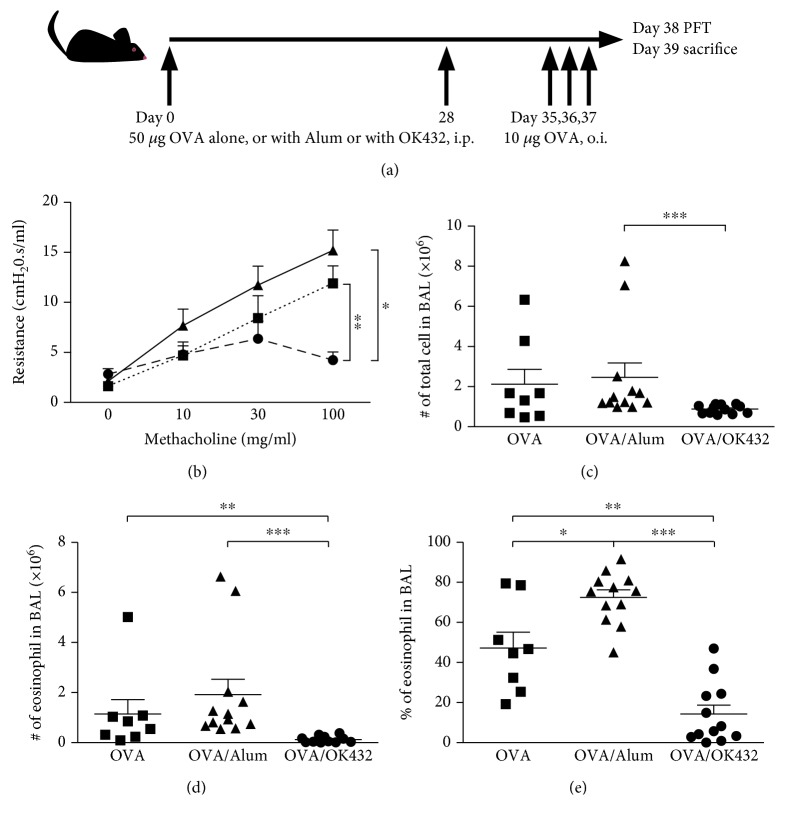
Pulmonary inflammatory responses induced by OVA challenge were diminished in mice preimmunized with OK-432 adjuvant. (a) Schematic diagram depicting protocols for immunization and challenge protocol. (b) Pulmonary function test was determined 24 hours after the last challenge. Mice were stimulated by increasing concentrations of methacholine (0, 10, 30, and 100 mg/ml), and airway resistances of different groups are shown. Cells in BAL fluid were stained by Liu's stain, and the number and percentage of leukocyte were calculated. Total cell number (c) and absolute number (d) and percentage of eosinophils (e) in BAL are shown. Data are represented as the mean ± SEM (*n* = 8–12 per group) from three independent experiments. ^∗^*p* < 0.05, ^∗∗^*p* < 0.01, and ^∗∗∗^*p* < 0.001.

**Figure 3:**
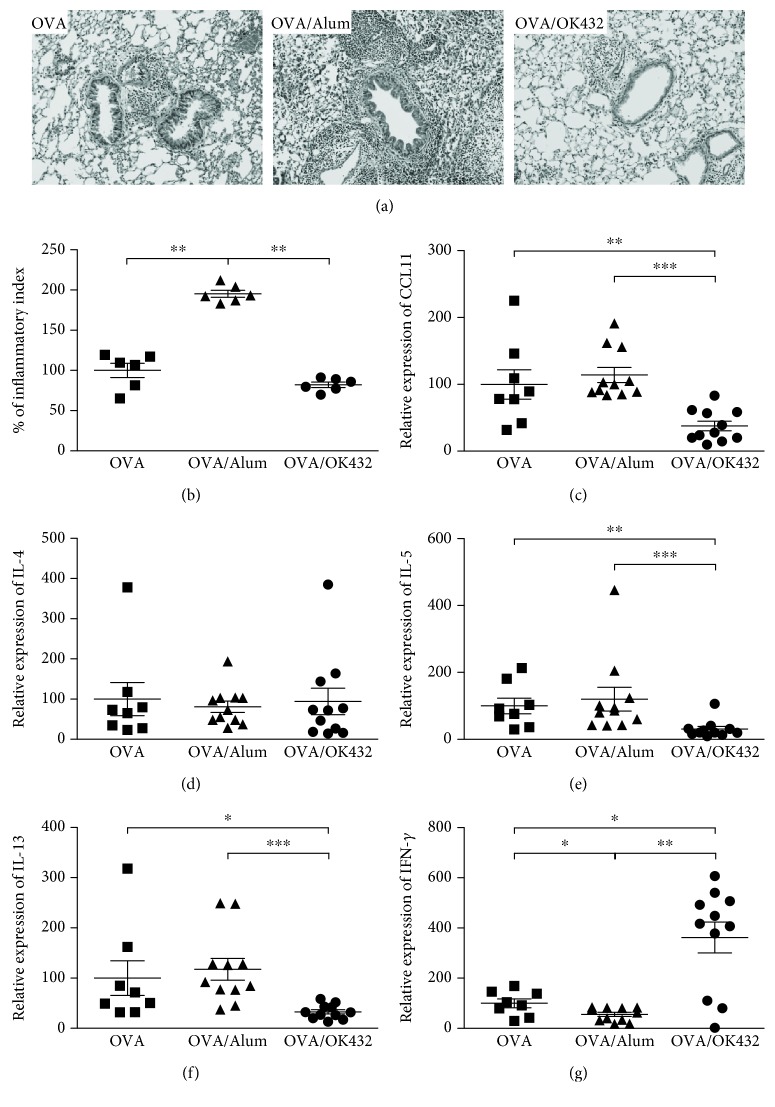
Histopathology shows less lung eosinophilia in mice preimmunized with OVA/OK-432 formula. (a) Formaldehyde-fixed, paraffin-embedded lung tissues from each group were sliced and stained with hematoxylin and eosin. One representative section at 200X is shown for each experimental group. Data are representatives of three independent experiments (*n* = 8–12). (b) Quantitative results of eosinophil infiltration (defined as inflammatory index) were analyzed by MetaMorph and expressed as percentage (mean ± SEM), compared to the OVA control mice. The mRNA levels of CCL11 (c), IL-4 (d), IL-5 (e), IL-13 (f), and IFN-*γ* (g) in lung tissues were determined by real-time PCR. Data are represented as the mean ± SEM (*n* = 8–12 per group) from three independent experiments. ^∗^*p* < 0.05, ^∗∗^*p* < 0.01, and ^∗∗∗^*p* < 0.001.

Thus far, we have shown that OK-432 can serve as an adjuvant during immunization to boost Th1 responses. However, it is unclear whether OK-432 could continue to enhance Th1-driven immune responses to subsequent antigen exposure at the anatomic site of natural antigen encounter. Of special interest is how OK-432-immunized mice respond when the same antigen is introduced to strictly lung tissue, an antigen-exposure route that strongly favors Th2 immune response [21]. Similar to our previous immunization protocol, mice were immunized and boosted with OVA alone (OVA) or in the presence of Alum (OVA/Alum) or OK-432 (OVA/OK-432). One week after boosting, mice were further challenged by oropharyngeal instillation (o.i.) for three consecutive days (Figure 2(a)). One day after the last challenge, the pulmonary function test was conducted to determine the airway resistance in response to increasing concentrations of methacholine. As shown in Figure 2(b), in response to OVA challenge by o.i., mice that had been immunized with OVA mixed with OK-432 exhibited significantly lower pulmonary resistance at 100 mg/ml of methacholine relative to mice immunized with OVA alone or with OVA mixed with Alum. In addition, OVA/OK-432 mice showed significant reductions in the total number of cells (Figure 2(c)) and the absolute number and the percentage of eosinophils (Figures 2(d) and 2(e), respectively) recovered by bronchoalveolar lavage (BAL) fluid, compared with OVA- and OVA/Alum-immunized mice. Consistent with a Th2-inducing adjuvant, the percentage of eosinophils in BAL was much higher in the OVA/Alum control than in the OVA without adjuvant control (Figure 2(e)). Histopathological analyses also revealed a marked reduction in eosinophils in the perivascular and peribronchial regions of the lungs from OVA/OK-432 mice and a large increase of lung eosinophilia from OVA/Alum controls (Figures 3(a) and 3(b)). Similarly, a significantly reduced mRNA level of CCL11, the most prominent chemokine for eosinophil trafficking [22], was detected in the lungs from OVA/OK-432-treated mice compared to controls (Figure 3(c)). Furthermore, lower Th2 cytokines and higher IFN-*γ* expression levels were also detected in lung tissues harvested from OVA/OK-432-treated mice relative to controls (Figures 3(d)–3(g)). Altogether, these results have demonstrated that inflammation caused by antigen challenge in a Th2-biased exposure route can be attenuated by prior vaccination with an OK-432 adjuvant.

### 3.3. Th1/Th2 Ratio Is Significantly Elevated in Mice Vaccinated with OVA/OK-432 Formula

**Figure 4:**
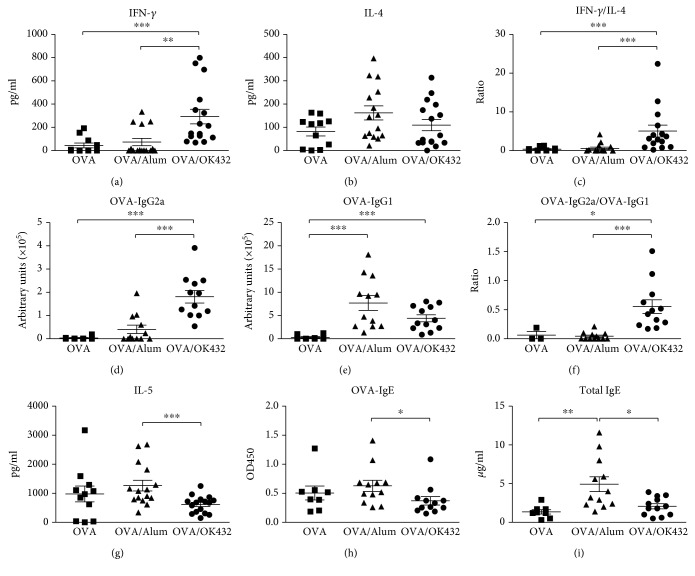
OVA/OK-432 formula induces more Th1 responses but controls Th2 reactions. The levels of IFN-*γ* (a), IL-4 (b), and IL-5 (g) in culture supernatants, in which spleen cells were restimulated by 100 *μ*g/ml OVA for 6 days in vitro, are shown. (c) IFN-*γ*/IL-4 ratios were calculated and depicted. The levels of OVA-IgG2a (d) and OVA-IgG1 (e) in sera were determined by ELISA. (f) OVA-IgG2a/OVA-IgG1 ratio from indicated mice was calculated and depicted. The titers of OVA-IgE (h) and total IgE (i) in sera from each group are shown. Data are represented as the mean ± SEM (*n* = 8–12 per group) from three independent experiments. ^∗^*p* < 0.05, ^∗∗^*p* < 0.01, and ^∗∗∗^*p* < 0.001.

To verify whether reduced pulmonary eosinophilia was caused by OK-432-enhanced Th1 responses in immunized mice, we next investigated the cytokine responses and OVA-specific Ab titers after antigen encounter. As shown in Figure 4(a), higher IFN-*γ* levels were detected in culture supernatants collected from OVA-stimulated spleen cells from OVA/OK-432 mice. On the other hand, OVA/Alum mice had a tendency to produce more IL-4 (Figure 4(b)). IL-5 has been shown to induce the differentiation, survival, and recruitment of eosinophils [23, 24]. In line with this, higher IL-5 production was detected in OVA-stimulated spleen cells from OVA/Alum control mice, in contrast to mice vaccinated with the OK-432 formulation (Figure 4(g)). Consequently, vaccination with OK-432 augmented IFN-*γ*/IL-4 cytokine ratios compared to a formulation with Alum adjuvant or antigen only (Figure 4(c)). Similarly, the antibody isotype profile in the serum of OVA/OK-432-immunized mice was consistent with a Th1 response, with high levels of OVA-specific IgG2a and low levels of anti-OVA IgG1 (Figures 4(d) and 4(e)). Therefore, the ratios of IgG2a/IgG1 antibody in the OVA/OK-432 group increased approximately ten-fold compared to those in the OVA/Alum control group (Figure 4(f)). Additionally, OVA-specific IgE, a major mediator in the allergic response, was higher in mice injected with the Alum formula than in those injected with the OK-432 formula (Figure 4(h)). A significant reduction of total IgE was also detected in the sera from mice treated with OVA/OK-432 compared with the OVA/Alum control group (Figure 4(i)). In summary, OK-432 can be used as a potent Th1 promoting and Th2 suppressing adjuvant for the prevention of Th2 bias immune responses.

## 4. Discussion

The purpose of prophylactic vaccination is to elicit suitable systemic immunity to protect individuals from disease. Vaccines are formulated with adjuvants to increase immune response, and the choice of adjuvant depends on the most appropriate type of immunity. Our findings have shown that OK-432 can act as an adjuvant to augment Ag-specific Th1 responses relative to Th2 bias adjuvant. Furthermore, we found that the development of airway hyperreactivity and Th2-related pulmonary inflammatory responses were inhibited in mice treated with an antigen OK-432 formula prior to allergen challenge. Finally, prophylactic treatment with OK-432 can dramatically reduce Th2-related humoral and cellular responses in the setting of OVA-induced asthma.

Toll-like receptors (TLRs) have been described as key molecules connecting innate and adaptive immunity [25]. It was confirmed that TLR4 signaling, not TLR2, is involved in OK-432-induced IFN-*γ* production and anticancer immunity [26]. Recently, Hovden and colleagues showed that TRIF-biased activation of TLR3 by OK-432 is responsible for the secretion of IL12 in human dendritic cells [27]. One possible explanation for the protective mechanism of OK-432 in allergic asthma is the induction of IFN-*γ* via TLR3/4 signaling. However, the involvement of TLR signaling in OK-432-enhanced Th1 responses needs to be further studied.

OK-432 impressively controls pulmonary eosinophilia as shown in Figure 2. Lower eosinophil counts correlate with lower CCL11 expressions in lungs isolated from OK-432-treated mice. Additionally, Fas-mediated apoptosis in eosinophils is believed to control the resolution of pulmonary Th2-driven allergic reaction [21, 28]. Recently, it was reported that the expression of perforin in cytotoxic CD8^+^ T cell is required to moderate allergic airway inflammation [29]. In line with this, the induction of perforin and enhanced expression of Fas-ligand are shown in OK-432-activated monocytes [30, 31], suggesting that an apoptosis-related mechanism might also be involved in controlling pulmonary eosinophilia by OK-432.

Current asthma therapies mainly treat symptoms of hypersensitivity after allergen stimulation. Allergen-specific immunotherapy represents a more targeted clinical approach to treating allergic diseases [32]. Unfortunately, at the moment, the high cost and risk of SIT-induced severe adverse side-effects, especially life-threatening anaphylactic shock, have limited its application. However, the possibility to vaccinate children with a genetic predisposition for developing allergies still exists. Notably, we found that the development of airway hyperresponsiveness and Th2-related humoral and cellular responses were inhibited in mice treated with the OK-432-modulated vaccine prior to allergen challenge.

Our results indicate that the adjuvant effect of OK-432 augments systemic Th1 bias response. Ag/OK-432 formulation attenuates the inflammation caused by antigen challenge in a Th2-biased exposure route. The ability to generate strong systemic Th1 activation in the presence of OK-432 adjuvant supports the particular relevance of applying this potential novel adjuvant for clinical use, especially in prophylactic vaccination for pathogens and immunotherapy in atopic diseases.

## 5. Conclusions

The adjuvant effect of OK-432 favors a systemic Th1 polarization that associates with increased IFN-*γ* and OVA-specific IgG2a. We found that mice treated with an antigen-OK-432 formula prior to allergen challenge did not develop airway hyperreactivity and Th2-related pulmonary inflammatory responses. Furthermore, Th2-related humoral and cellular responses in the setting of antigen-induced asthma could be dramatically reduced by the prophylactic treatment with OK-432. The ability to generate strong systemic Th1 activation in the presence of OK-432 adjuvant supports the potential adjuvant for clinical use.

## Data Availability

The data used to support the findings of this study are available from the corresponding author upon reasonable request.
